# Mr.Vc: a database of microarray and RNA-seq of *Vibrio cholerae*

**DOI:** 10.1093/database/baz069

**Published:** 2019-06-24

**Authors:** Zhiyuan Zhang, Guozhong Chen, Jun Hu, Wajid Hussain, Fenxia Fan, Yalin Yang, Zhigang Zhou, Xiaodong Fang, Jun Zhu, Wei-Hua Chen, Zhi Liu

**Affiliations:** 1 Department of Biotechnology, College of Life Science and Technology, Huazhong University of Science and Technology, Wuhan 430074, China; 2 State Key Laboratory of Infectious Disease Prevention and Control, National Institute for Communicable Disease Control and Prevention, Chinese Center for Disease Control and Prevention, Beijing 102206, China; 3 Sino-Norway Fish Gastrointestinal Microbiota Joint Lab, Feed Research Institute, Chinese Academy of Agricultural Sciences, Beijing 100081, China; 4 The Second Affiliated Hospital of Guangzhou University of Chinese Medicine, Bioinformatics Group, The Second Affiliated Hospital of Guangzhou University of Chinese Medicine, Guangzhou 510120, China; 5 College of Life Science, Henan Normal University, Xinxiang 453007, China; 6 Huazhong University of Science and Technology Ezhou Industrial Technology Research Institute, Ezhou, Hubei 436044, China

## Abstract

Gram-negative bacterium *Vibrio cholerae* is the causative agent of cholera, a life-threatening diarrheal disease. During its infectious cycle, *V. cholerae* routinely switches niches between aquatic environment and host gastrointestinal tract, in which *V. cholerae* modulates its transcriptome pattern accordingly for better survival and proliferation. A comprehensive resource for *V. cholerae* transcriptome will be helpful for cholera research, including prevention, diagnosis and intervention strategies. In this study, we constructed a microarray and RNA-seq database of *V. cholerae* (Mr.Vc), containing gene transcriptional expression data of 145 experimental conditions of *V. cholerae* from various sources, covering 25 937 entries of differentially expressed genes. In addition, we collected relevant information including gene annotation, operons they may belong to and possible interaction partners of their protein products. With Mr.Vc, users can easily find transcriptome data they are interested in, such as the experimental conditions in which a gene of interest was differentially expressed in, or all genes that were differentially expressed in an experimental condition. We believe that Mr.Vc database is a comprehensive data repository dedicated to *V. cholerae* and could be a useful resource for all researchers in related fields. Mr.Vc is available for free at http://bioinfo.life.hust.edu.cn/mrvc.

## Introduction

Cholera is a notorious diarrheal disease, which caused great epidemic seven times throughout the world in history, and is still endemic in many parts of the world, especially developing countries in Asia, South America and Africa (1, 2). To date, 1.3 to 4 million cases of cholera occur annually with 23 000 to 143 000 deaths (3). Cholera is a major public health problem (4), particularly in regions with poor socioeconomic condition and sanitation (5). Cholera epidemiology is closely associated with aquatic ecology of its causative agent, *Vibrio cholerae* (6, 7). *V. cholerae* is a waterborne bacterium often exists in aquatic environment, such as seas, rivers, ports, estuaries and pond waters. During infection, *V. cholerae* passages through gastric acid in the stomach and colonizes on the epithelial cell surface of small intestine. For better survival and infection, *V. cholerae* quickly modulates its gene transcriptional expression in response to the switches of different environments.

**Figure 1:**
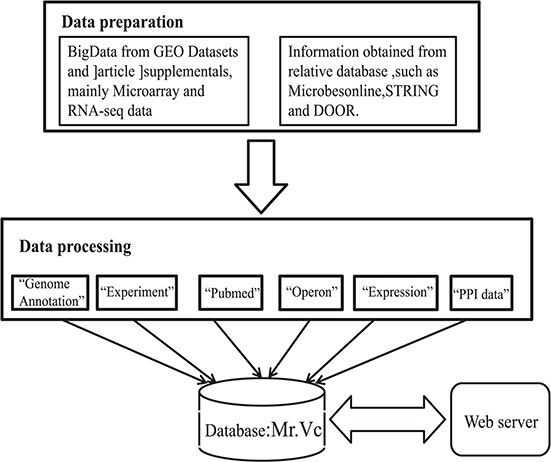
Data acquistition and organization in Mr.Vc database.

Microarray and RNA-seq are powerful techniques to study general gene expression profiles. There have been many reported microarray and RNA-seq data of *V. cholerae* transcriptomic change in response to various environmental stimuli including serine hydroxamate (8), bile (9), stress (10, 11) and in different gene deletion background, such as *rpoN* (12), *rpoH* (13), *cgtA* (8), *cpxR* (14), *nqrA* (15). However, those important transcriptome data have been uploaded separately to various databases, such as Microbesonline (www.microbesonline.org) (16), DOOR (www.csbl.bmb.uga.edu/DOOR) (17), STRING (http://string-db.org) (18), which creates obstacles for cholera researchers to have a comprehensive access to these data.

**Figure 2:**
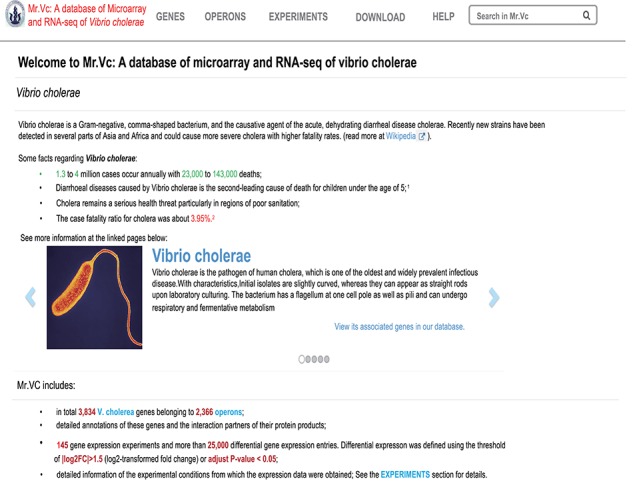
Interface of Mr.Vc database homepage.

**Figure 3:**
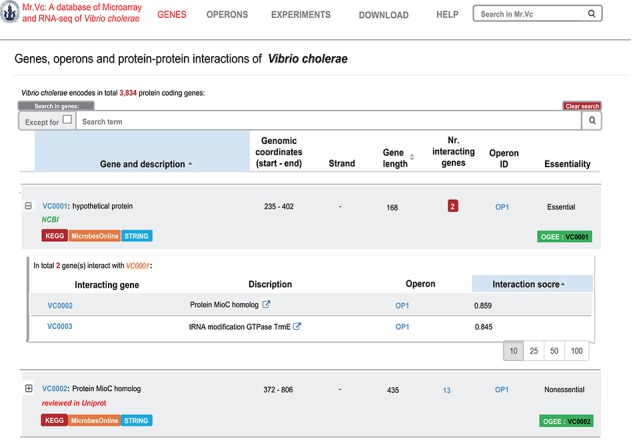
‘‘GENES’’ page of Mr.Vc database.

To make it more efficient and pain-free for researchers to obtain all *V. cholerae* data in a centralized database, we constructed Mr.Vc, a comprehensive database of microarray and RNA-seq data of *Vibrio cholerae*. In Mr.Vc, we collected data from 145 high-throughput gene expression experiments of *V. cholerae* from 49 journal articles. In addition to the detailed annotation for 3834 *V. cholerae* genes, we also collected relevant information including which operons they may belong to and possible interaction partners of their protein products. To our knowledge, Mr.Vc is the first database dedicated for transcriptome data for *V. cholerae.*

## Materials and methods

### Database construction

For initial literature screening, we retrieved 11 705 articles and related information from the PubMed website (https://www.ncbi.nlm.nih.gov/pubmed) with a query ‘*Vibrio cholerae*’ [ALL Fields]. We further filtered the above articles using ‘microarray’, ‘transcription profile’, ‘transcriptome’, ‘RNA-seq’ or ‘high throughput’ and obtained 251 records. We downloaded and curated all records manually, and finally identified 49 original research articles with sufficient *V. cholerae* transcriptome data. The workflow of literature mining and manual curation was shown in Figure 1, and the database will be updated with newly published articles in *V. cholerae* research.

**Figure 4:**
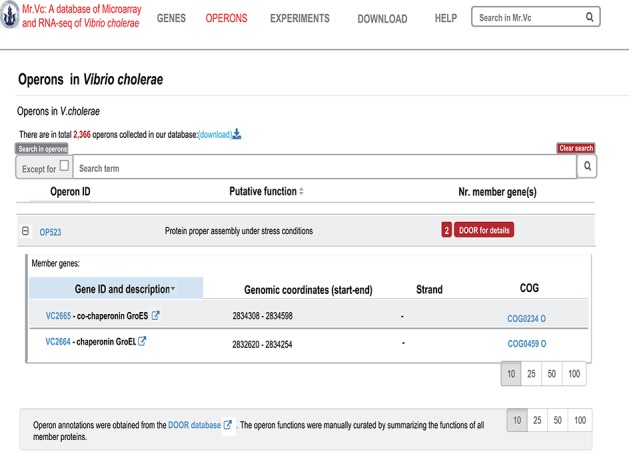
‘‘OPERONS’’ page of Mr.Vc database.

**Figure 5:**
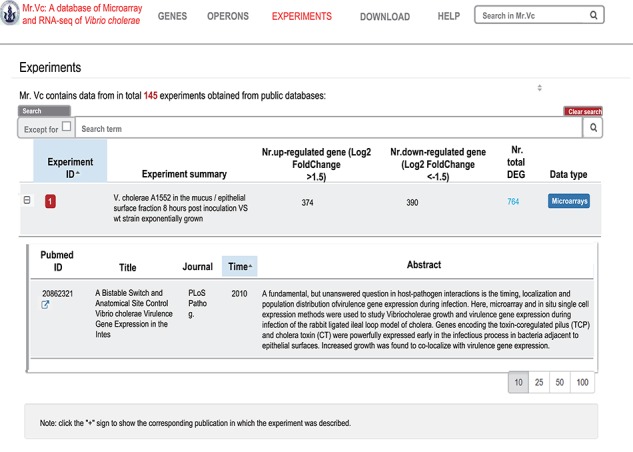
‘‘EXPERIMENTS’’ page of Mr.Vc database.

We downloaded all expression data from the NCBI GEO database (www.ncbi.nlm.nih.gov/geo/) (19). In total, we obtained expression data from 54 microarray experiments, all of which used two-channel microarrays, in which with gene expression levels shown as fold changes, which are not comparable across different experiments. To make the expression data can be compared across experiments, we downloaded raw signal intensity values for all the 54 experiments, treated the two-channels of the microarray data as independent experiments and used an in-house R script to do the normalization. The normalized intensity values can be compared across any experimental conditions. For microarray experiments, we used a cutoff of |log_2_ FC|>1.5 (FC, fold change) to define differentially expressed genes between experiment and control conditions. Please note that this cutoff may have different meanings for genes with different expression abundances. For example, due to technical limitations and/or random fluctuation, the expression abundances of lowly expressed genes under different conditions can easily differ more than 1.5 fold. The two-channel array experiments lacked technical/biological replicates, which made it impossible to compute *P* values by ourselves. To circumvent these shortcomings, we decided to adopt a rather stringent cutoff of |log_2_FC|>1.5 rather than the commonly used 1 in our database.

We also obtained 31 RNA-seq data sets, in which the expression abundances were normalized as RPKM (reads per kilobase per million sequences) values. We used differentially expressed genes obtained from the literature, which often came with *P* values to indicate whether the differences are significant or not. Genes with *P* values <0.05 were considered as differentially expressed genes.

A total of 25 937 different gene expression entries were extracted, representing *V. cholerae* gene expression under 145 different experimental conditions. All were listed in ‘expression’ table; the information of corresponding experiments were listed in the ‘experiment’ table.

We compiled gene information including gene IDs, gene official names, descriptions and genomic locations for all of the 3834 *V. cholerae* genes, by pulling information from NCBI RefSeq and UniProt (20) databases. We obtained operon annotations from the DOOR database (17). We included links to external databases including KEGG (21) and Microbesonline (16), from which users can get metabolic genes and pathways of *V. cholera*e, the STRING database (18) in which protein–protein interaction information are available, the OGEE database (22) in which gene essentiality information can be obtained. These information can give researchers more clues about how *V. cholera*e modulates gene regulation.

All the above information can be downloaded from the ‘Download’ page either separately or together as a database dump file in SQL format.

### Database design

Mr.Vc was designed as a relational database on an Apache server of XAMPP, which integrated MySQL database, Apache and Tomcat for convenience. All extracted data from published journal articles or databases were organized in an available MySQL database as the back end, along with a user-friendly graphical interface based on CSS, HTML and JavaScript as the front end. PHP scripts were used to generate HTML web pages. In addition, the database administration tool was phpMyAdmin 4.7.4, which is used for data entry.

### Implementation

Users can browse through the database content or search specific topics by inputting keywords for search request. Search requests would be sent to a PHP script that handles communications between the users and servers. The PHP script sends the search request to a MySQL database for retrieving desired information. Finally, data return to web surface to display. JavaScript and CSS were used for the user-interface of the web pages.

## Result

### Database content

To date, Mr.Vc database documents 25 937 gene expression data of *V. cholerae* under 145 different experiment conditions, including 2 serotypes (classical and El Tor strain), 3834 genes, 2366 operons and 67 988 protein–protein interactions. For each gene, in addition to transcriptional expression changes, other relevant details are also provided, such as gene locus ID, gene official name, its location on the genome, description, operon that it belongs to and its putative protein–protein interaction partners available from public databases.

### Web interface

The Mr.Vc website consists of four main functional modules including ‘Genes’, ‘Operons’, ‘Experiments’, ‘Downloads’ (Figure 2), allowing users to browse, search and download all Mr.Vc data and related information. The main purpose of the web interface design is to help researchers quickly access expression profiles of genes of interest in *V. cholerae* and search for contents they are interested in. A global search widget enables users to search any information by gene IDs, names or experiment IDs. Links to external databases were included in Mr*.*Vc, allowing users to find additional useful information in other public databases*.* To give users a clear overview of the data contents, their organization in our database, functionalities of our database and the usage, we provided detailed information about Mr.Vc in the ‘Help’ section. Mr*.*Vc also has a feedback option. Users can email the authors about any problems they encounter.

### Genes

The ‘Genes’ page shows all the individual gene information, including gene ID, description, gene location, gene orientation, gene length and gene essentiality (Figure 3). We also report here associated genes and the operon information, allowing users to find the regulation information of their target gene. In addition, links to external databases including NCBI, KEGG (21), Microbesonline (16), STRING (18) and OGEE (22) were also included, allowing users to explore in more details of these gene in those public databases.

### Operons

In the ‘Operons’ page (Figure 4), users can find a list of all operons of *V. cholerae*, their member genes and the putative operon functions summarized from all the members. Operon annotations were obtained from the DOOR database (17). For each member of the operon, we report the gene location, orientation and a brief annotation. The ‘operon ID’ tab leads to more detailed operon information, and the ‘Numbers of the operon member’ link leads to more information on the gene members of the operon. User can directly type in the target gene name or ID in the search area to browse more information of the corresponding operon.

### Experiments

The ‘Experiments’ page (Figure 5) is an exhaustive list of all the transcriptome experiments, 145 currently, in the database. On this page, a table was used to provide a summary report on each experiment, including the experimental ID, brief summary of the experimental condition, numbers of up- and down-regulated genes (differentially expressed genes, DEGs) and the methods type (microarray or RNA-seq). Users can expand the table by clicking the ‘+’ sign before the ‘Experimental ID’ to view more details on the experimental design and the corresponding reference(s); by clicking the ‘Total DEGs’ link, users will be redirected to the complete list of the DEGs of the corresponding experimental condition.

### Downloads

**Figure 6:**
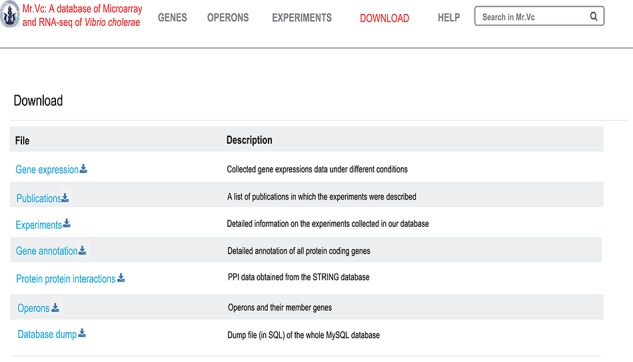
‘‘DOWNLOAD’’ page of Mr.Vc database.

All Mr.Vc entries are downloadable as excel files at the ‘Downloads’ page (Figure 6).

## Discussion

The Mr.Vc database has collected all the *V. cholerae* transcriptome profiles (145 by far) from the published literature, the largest and most comprehensive specialized database to date; as comparison, Microbesonline (16), which integrated vast amounts of microbial genetic information, has only 42 high-throughput *V. cholerae* transcriptome data under different experimental conditions, deriving from seven published papers.

We believe that Mr.Vc will be a powerful tool for researchers in cholera and related fields. In the Mr.Vc database, users can quickly access gene expression profiles in *V. cholerae* under published experimental conditions by a simple search with a gene ID or name, with genes that are differentially transcribed under the same condition showing up as additional information.

*V. cholerae* is an important pathogenic bacterium and a model organism for studying the molecular mechanisms of pathogenesis. Our Mr.Vc database will facilitate thousands of *V. cholerae* researchers all over the world. Currently, the Mr.Vc database includes more than 25 000 DEG entries identified using microarray or RNA-seq data. These data were extracted from 145 high-throughput gene expression experiments published in 49 research papers. Researchers can also get the original information by clicking on the relevant hyperlink to PubMed and can easily download relevant information such as annotation, operon, gene location, etc. The Mr.Vc database provides links to other databases, for example, DAVID and KEGG, that will allow researchers to access the gene regulation networks and other aspects of the target genes.

Operons, as the basic function unit of the genome, are fundamental subjects when examining gene transcriptional expression. People have developed many platforms for bacterial operon research, such as DOOR ‘the Database of prOkaryotic OpeRons’ (17). We integrated *V. cholerae* operon information into the Mr.Vc database. Mr.Vc users can easily find the gene operon data, and may seek the published literature related to any of the genes in this operon, through the embedded hyperlinks.

Proteins are the products of gene expression. In pathogenic bacteria, proteins not only participate in cell metabolism, constitute cell structures, but can also be the disease causative toxin, such as cholera toxin. ‘STRING’ (18) and other databases provide massive information of proteins and protein–protein interactions of thousands of organisms. For *V. cholerae* researchers interested in protein data, the Mr.Vc database incorporated about 100 000 information of *V. cholerae* protein and protein–protein interactions from STRING.

In the future, we will continue to update microarray and RNA-seq data extracted from the growing body of literature. We hope that our Mr.Vc database can help researchers in the *V. cholerae* and related fields with more convenient and comprehensive information of *V. cholerae* transcriptome.

## Acknowledgements

We greatly thank Dr Yitian Zhou (University of Pennsylvania, USA) and Dr Balakrishnan Subramanian (Huazhong University of S&T) for critical review of the manuscript.

## Notes

**Database URL**: http://bioinfo.life.hust.edu.cn/mrvc
